# On the difficulty of achieving Differential Privacy in practice: user-level guarantees in aggregate location data

**DOI:** 10.1038/s41467-021-27566-0

**Published:** 2022-01-10

**Authors:** Florimond Houssiau, Luc Rocher, Yves-Alexandre de Montjoye

**Affiliations:** 1 Department of Computing, Imperial College London, SW7 2AZ London, UK; 2 Alan Turing Institute, 2QR, John Dodson House, 96 Euston Rd, London, NW1 2DB UK; 3 Oxford Internet Institute, University of Oxford, OX1 3JS Oxford, UK

**Keywords:** Computational science, Social sciences

**arising from** A. Bassolas et al. *Nature Communications* 10.1038/s41467-019-12809-y (2019)

While large-scale human mobility data contain crucial insights to understand human behavior, they are also highly sensitive. Google shared with Bassolas et al. anonymous aggregated data from 300M Google Maps users and stated that sharing this dataset raises no privacy concern as this data would “at best improve the level of certainty [of an attacker to infer if a user is in the dataset] over a random guess by approximately 16%”. We believe these guarantees rely on assumptions that are not met in practice. Using their attack model on a real-world mobility dataset, we instead argue that the level of certainty of their attacker is likely higher than 90% for a typical user (e.g., 95.4% for one of the authors who made 32 unique trips in a week). Weak anonymization methods and unrealistic privacy guarantees have eroded public trust in the past. As new anonymization methods are being deployed, we ought to ensure that the risks and the guarantees they give are correctly communicated to data subjects.

The dataset used by Bassolas et al.^1^ consists of aggregated end-to-end trips, count matrices where each entry is the number of people who traveled from one location to another at least once during a given week (unique trips for a user). Laplace noise with zero mean and scale 1/*ε* is added to the counts with an *ε* parameter of 0.66. Origin-destination cells with user count lower than 100 people (after noise addition) are removed. In their article, the authors state that this “yields (*ε*, *δ*)-differential privacy guarantee of *ε* = 0.66 and *δ* = 2.1 × 10^−29^”, and conclude that sharing the dataset does not improve the level of certainty of a strong attacker by more than 16%.

To draw this conclusion, Bassolas et al. use the standard differential privacy membership attack model: a strong attacker having access to all the records in the dataset, except the victim’s, and auxiliary information about him or her. The 16% certainty bound they report, however, relies on the assumption that any one user does not contribute more than one trip to the dataset. While Bassolas et al. do not report the total number of trips, the analyses performed in the paper strongly suggest that users contribute substantially more than one trip to the dataset.

## Results

**Fig. 1:**
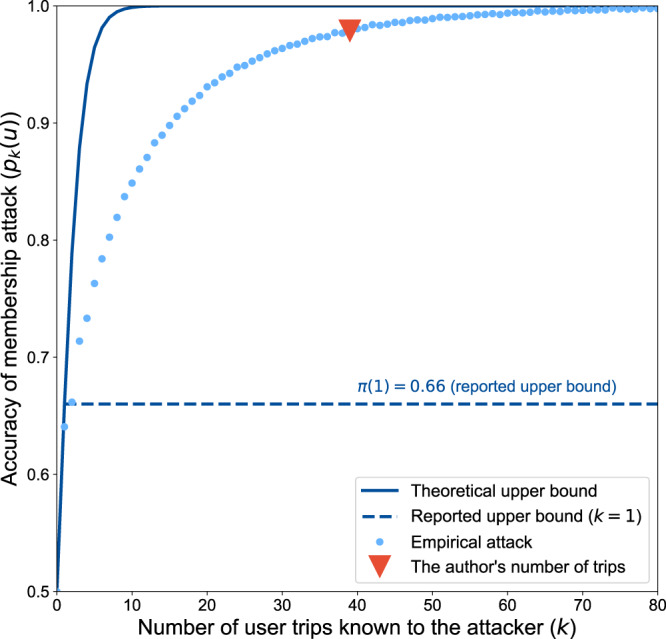
The accuracy of the attack increases steeply with attacker knowledge and is higher than the reported upper bound. Empirical accuracy of a membership attack (dots) and theoretical upper bound (solid line) as a function of the number of unique trips taken by a user. Reported upper bound (dashed line) and empirical accuracy of the attack on one of the authors’ data (triangle).

Using Bassolas et al.’s attack model on a real-world mobility dataset, we show the empirical risk to be higher than the 16% bound as soon as the victim took more than three unique trips over any week (*p*_3_(*u*) = 70.5%). Figure 1 reports the accuracy of the membership attack, *p*_*k*_(*u*), the likelihood that an attacker can test if *u* is in the aggregated data knowing *k* trips from their trajectory. The average number of trips per user in Bassolas et al.’s dataset is not reported but, looking at the Google Maps Timeline^2^ of one of us, the 39 trips taken over a typical week with 32 of them being unique would give an attacker a 95.4% certainty that he is in the dataset.

From a differential privacy standpoint, the guarantees given by the authors (*ε* = 0.66) protect single trips taken by a victim. A conservative estimate of the total privacy loss for *any user* in the dataset for 1 week, obtained through a straightforward application of the composition theorem, would be *ε* = 46.2 and *δ* = 2.1 × 10^−29^ (see “Methods”). Standard values of *ε* range from “*as little as 0.01 to as much as 7*” in the literature^3^, with higher values providing exponentially weaker guarantees. This privacy loss would furthermore grow linearly with the number of weeks of data released; for the 1 year of data released here, the *ε* budget could be as much as 52 times higher.

We believe that behavioral data have a great potential for good, especially in research, and that data should be used a lot more than they are currently. However, in the pursuit of this laudable goal, we have allowed weak de-identification methods relying on unrealistic assumptions to be used to anonymize data behind closed doors. The guarantees given by these methods were incorrect in practice, leading numerous datasets to be re-identified and eroding public trust^4–6^.

The standard attack model considered by the authors is very strong and probably not realistic in a setup where aggregated data is shared with trusted researchers. However, as we are moving away from de-identification and towards modern privacy engineering solutions such as Differential Privacy, it is crucial for us as a community to learn from previous mistakes. The inherent risks and limitations of data sharing have to be clearly communicated and the guarantees we give data subjects correct and transparent. The future and broad availability of data for research are at stake.

## Methods

### Contribution of each user to the dataset

The guarantees given by Bassolas et al. rely on the assumption that each user does not contribute more than one trip to the dataset. The analyses performed in the paper strongly suggest that users contribute more than one trip to the dataset.

Indeed, the final dataset used by Bassolas et al. contains connections, origin-destination trip counts, with at least 100 trips each. The dataset contains for instance 46,333 connections for Atlanta (population 5 M). Assuming that each user reports exactly one trip, at least 4.6 M people from Atlanta should contribute to the dataset to get the number of connections reported in the dataset. Given that only 67% of US mobile phone users use Google Maps as their main navigation app^7^, we find this unlikely, thereby strongly suggesting that some users contributed more than one trip to the dataset.

Regarding unique trips, the authors later confirmed that each user contributes a list of their unique weekly trips to the weekly aggregate. If the same trip (A → B) is made several times in a week, it is only counted once. In the case of one of the authors having made 39 trips in a given week, this results in him contributing 32 unique trips to the weekly aggregate while 7 of them would be discarded. Note that here unique refers to trips that are unique for a given user during a given week.

### Generating trips from empirical data

We use a longitudinal mobility dataset extracted from CDR data. Each individual trajectory contains points with time and approximate location (antennas). We segment trajectories using a winner-takes-all approach, selecting the most used location for every hour, and define a trip as a movement from one location to a distinct one in the consecutive hour.

### Performing the attack

We follow the procedure described by Bassolas et al. to aggregate anonymized trips: computing the origin-destination count matrix for unique trips, add zero mean Laplacian noise with scale 1/*ε* to each entry, and discard all (noisy) counts lower than 100.

We then use the attack model the authors rely on to compute the 16% increase over a random guess: the standard membership inference attack with perfect knowledge. In this model, the strong attacker has access to all the records in the dataset, except the victim’s, and auxiliary information about the victim.

More specifically, for *k* between 0 and 70, we select one user *u* with exactly *k* trips. The attacker performs a membership attack to test whether the anonymized data *D*′ they received is *D*^+^ (the anonymized trajectories with *u* included) or *D*^*−*^ (without *u* included). We compute the local origin-destination matrix *A*(*u*) for the user *u* and, by linearity of the noise addition, compute the normalized matrix *A*(*D*′) *- A*(*D*^*−*^) generated from either no user or *u*. We perform a likelihood-ratio test to distinguish whether the normalized matrix was sampled from a Laplacian distribution *L*(0, 1/*ε)* or *L*(*A*(*u*), 1/*ε*).

We repeat this procedure 10,000 times for all values of *k* between 0 and 70 and report the mean in Fig. 1.

### Theoretical bounds

The theoretical bound reported by Bassolas et al. is obtained by bounding the posterior probability of an attacker trying to infer whether a user is in the dataset, *π*(*y*). Formally, let D* be the tested dataset, *D*^+^ the dataset with the user *u*, and *D*^−^ the data without *u*. If the attacker’s prior holds no information (e.g., when *P*[D* = *D*^+^] = 0.5), we then have for all *y* (and for *M* an ε-DP mechanism^8^):

$$\frac{\pi (y)}{1-\pi (y)}=\frac{P[{D}^{\ast }={D}^{+}|M({D}^{\ast })=y]}{P[{D}^{\ast }={D}^{-}|M({D}^{\ast })=y]}=\frac{P[M({D}^{+})=y]}{P[M({D}^{-})=y]}\le {e}^{\varepsilon }$$

which then implies *π*(*y*) *≤* *e*^*ε*^/(1 + *e*^*ε*^).

### Conservative estimate of the privacy loss

To estimate the privacy loss for any user in 1 week of data, we assume conservative bounds: each user contributes only once to each count and makes no more than 70 unique trips per week (10 per day). Let *n*_trips_ be the maximum number of unique trips that any user could contribute to the data, then the L_1_ sensitivity of the count matrix is *n*_*t*rips_. Adding Lap(1/*ε*) noise and low-count filtering implies (*n*_trips_ × *ε*, 2.1 × 10^−29^)-differential privacy by a straightforward application of simple composition bounds^9^.

Similarly, the privacy loss for a year of data can be estimated as the sum of the privacy losses for every week. A reasonable estimate of the total loss of the data release would thus be 52 times the privacy loss for a week, *ε*_total_ = 52 × *n*_trips_ × *ε* = 2402.4.

Note that, while better bounds can be obtained, they require larger values of *δ*^8,9^. In this specific case, acceptable values of *ε* would require prohibitively large values of *δ* rendering the guarantees meaningless in practice.

## Data Availability

Although the raw data used in our experiments cannot be shared for confidentiality reasons, a synthetic mobility dataset that closely replicates the findings is available on request from the authors. Similarly, the source data for Fig. 1 is available on request from the authors.

## Code availability

The code to perform our attack is available from the corresponding author upon reasonable request for academic purposes.
